# *Drosophila* miR-33-5p Suppresses Cell Growth by Inhibiting ERK Signaling

**DOI:** 10.3390/biology14121693

**Published:** 2025-11-28

**Authors:** Taeheon Lee, Nayeon Kim, Ye Jin Park, Seungeun Cha, Young Sik Lee, Do-Hwan Lim

**Affiliations:** 1 College of Life Sciences and Biotechnology, Korea University, Seoul 02841, Republic of Korea; taehun1114@korea.ac.kr; 2 School of Systems Biomedical Science, Soongsil University, Seoul 06978, Republic of Korea; kno0122@naver.com (N.K.); glory01kr@soongsil.ac.kr (Y.J.P.); gndgnddl1@naver.com (S.C.); 3 AI-BIO Convergence Research Institute, Soongsil University, Seoul 06978, Republic of Korea

**Keywords:** *Drosophila*, miR-33, *Ras64B*, p-ERK, growth

## Abstract

Cell growth control is a critical process underlying diverse biological events, including survival, development, tissue repair, and disease. Growth regulation is orchestrated by a combination of external and internal cues, involving a multitude of signaling pathways. Nevertheless, our comprehension of the regulation of growth-associated signaling pathways is still incomplete. In this study, we discovered that microRNA *miR-33* overexpression in *Drosophila* S2 cells resulted in a reduction in cell proliferation. This growth inhibition was attributed to the inactivation of ERK signaling, which is mediated through *Ras64B*, a direct target of miR-33-5p. In accordance with these observations in S2 cells, miR-33 inactivation in *Drosophila* wings led to an increase in cell number, while its overexpression resulted in a decrease. Notably, miR-33-induced wing reduction was associated with diminished ERK signaling, and this wing defect was rescued by co-expression of *Ras64B* or a constitutively active ERK variant. Consequently, these findings establish miR-33–*Ras64B*–ERK as a regulatory axis, providing new mechanistic insights into growth control in *Drosophila*.

## Simple Summary

Cell and tissue growth is a fundamental biological process essential for the survival of an organism. Accordingly, elucidating the mechanisms that finely regulate growth is of great importance. However, because these processes involve complex interactions among diverse molecular factors, they are not yet fully understood. In this study, we identified miR-33, a small non-coding RNA that regulates gene expression and is associated with cell growth in *Drosophila*. In *Drosophila* S2 cells, *miR-33* expression inhibited cell proliferation and increased cell death. This growth suppression was mediated through the targeting of *Ras64B*, ultimately resulting in reduced ERK signaling. Similarly, in *Drosophila* wings, *miR-33* expression decreased the overall wing size by reducing the cell number, and the phenotype is also associated with *Ras64B*-mediated downregulation of ERK signaling. Taken together, our findings reveal a novel miR-33–*Ras64B*–ERK regulatory axis that controls cell and tissue growth in *Drosophila*.

## 1. Introduction

Cellular growth is regulated by several cellular processes, primarily determining cell mass accumulation, cell division, and cell survival [1]. These processes collectively influence cell size and number; cell mass accumulation affects cell size, while cell division and cell survival primarily affect cell number. During *Drosophila* wing development, multiple signaling pathways contribute to cell growth regulation, particularly those controlling cell number, including the Wingless signaling pathway [2], the Hippo signaling pathway [3], and the EGF/Ras signaling pathway [4].

In the EGF/Ras signaling pathway, Ras activation is initiated upon EGF binding to receptor tyrosine kinases (RTKs), leading to the propagation of signals to crucial downstream effectors such as Raf kinases and phosphatidylinositol-3 kinase (PI3K) [5]. Notably, distinct Ras isoforms exhibit varying preferences for downstream effectors. K-Ras preferentially activates Raf-1, while H-Ras is more likely to engage PI3K [6]. R-Ras, a Ras-related protein family, is named for its homology to the classical Ras family. It comprises three members: R-Ras1, R-Ras2 (TC21), and R-Ras3 (M-Ras) [7]. In *Drosophila*, two Ras isoforms are present: Ras oncogene at 85D (*Ras85D*; *Ras1*) and Ras oncogene at 64B (*Ras64B*; *Ras2*). *Ras85D* is homologous to classical Ras, whereas *Ras64B* is more closely related to TC21.

Several studies investigated the role of *Ras85D* in *Drosophila* wing development, demonstrating its crucial role in regulating cell proliferation by promoting the G1/S transition [8]. Notably, these effects are primarily mediated through the Raf/MEK/ERK signaling cascade rather than the PI3K signaling cascade [9,10]. In contrast, *Ras64B* remains relatively understudied, although its overexpression has been reported to induce excessive vein formation [11].

MicroRNAs (miRNAs) are approximately 22 nucleotides long, non-coding RNA molecules that play a pivotal role in post-transcriptional gene regulation [12]. Initially, transcribed primary miRNAs are processed into precursor miRNAs by the Drosha-DGCR8 complex, and then cleaved into miRNA duplexes by Dicer [13]. Of these duplexes, only one strand (either the -5p or -3p strand, derived from the 5′-arm or 3′-arm of precursor miRNA, respectively) functions as gene regulator. In *Drosophila*, miRNAs function by interacting with complementary sequences in the 3′-untranslated regions (3′-UTRs) of target mRNAs after the assembly of the miRNA-induced silencing complex (miRISC), which contains the key protein Argonaute-1 (Ago1) [13]. This interaction typically leads to the suppression of protein translation or the degradation of target mRNAs. Large-scale small RNA sequencing analyses identified a total of 469 mature miRNAs in *Drosophila* [14]. However, the biological functions of only a limited subset have been elucidated.

Among these miRNAs, several have been reported to regulate the activity of the insulin/insulin-like growth factor signaling (IIS) pathway, a crucial pathway associated with *Drosophila* developmental growth. For instance, miR-14 regulates IIS by suppressing sugarbabe (*sug*), a gene involved in *Drosophila* insulin-like peptide (dILP) production and secretion [15]. miR-276-3p negatively regulates IIS by directly targeting Insulin-like receptor (*InR*) [16]. miR-263b-5p also functions as a negative regulator of IIS by directly targeting Akt kinase (*Akt*), a core component of the pathway [17]. Conversely, miR-8 enhances IIS by targeting u-shaped (*ush*), a negative regulator of PI3K [18]. In contrast, the functional characterization of miRNAs involved in regulating Ras signaling, another key growth-related pathway in *Drosophila*, remains largely unexplored. To date, only the miR-279/996 cluster has been reported to regulate Ras signaling by directly targeting rhomboid (*rho*) and roughoid (*ru*), two positive regulators of the pathway, thereby contributing to eye development [19].

Previous studies have shown that miR-33 acts a key regulator of lipid metabolism by targeting multiple target genes, including *atpcl*, *midway*, and *Akt1*, which are involved in fatty acid synthesis and degradation [20]. As an extension of this study, we identified a novel regulatory network of *Drosophila* miR-33-5p in the control of cell growth. Overexpression of *miR-33* in *Drosophila* S2 cells resulted in diminished cell proliferation and augmented cell death. These phenotypic alterations induced by *miR-33* overexpression were associated with *Ras64B*, an identified target gene of miR-33-5p. In accord with the results of *miR-33* overexpression, depletion of *Ras64B* led to a reduction in cell proliferation and an enhancement of cell death. Furthermore, *Ras64B* depletion resulted in diminished ERK signaling activity, as evidenced by reduced p-ERK levels. Notably, the suppression of ERK signaling through the interaction between miR-33-5p and *Ras64B* also contributed to reduced cell proliferation during *Drosophila* wing development. Collectively, these findings demonstrate that miR-33-5p regulates cell growth by targeting *Ras64B* and modulating ERK signaling, thereby extending the biological role of miR-33 to the Ras/ERK signaling pathway beyond metabolic regulation.

## 2. Materials and Methods

### 2.1. Cell Culture and Transfection

*Drosophila* S2 cells were cultured in Schneider’s insect medium (Welgene, Gyeongsan, Republic of Korea) supplemented with 10% fetal bovine serum (Welgene) and 100 U/mL penicillin–streptomycin (Welgene) at 25 °C. S2 cells (1.4 × 10^6^ cells per well in a 12-well plate) were seeded in well plates. DNA constructs or double-strand RNAs (dsRNAs) were transfected into S2 cells using the CalPhos Mammalian Transfection Kit (Takara Bio, Kusatsu, Japan) or the TransIT-Insect Transfection Reagent (Mirus Bio, Madison, WI, USA), according to the manufacturer’s instructions. For 12-well plate transfections, 1 mg of DNAs or 6 mg of dsRNAs were used per well. For experiments utilizing the pMT vector, CuSO_4_ was added to the culture medium at a final concentration of 1 mM to induce expression of the inserted gene. Analyses were conducted 48 h or 72 h after induction with CuSO_4_ or post-transfection with dsRNA. Specifically, expression changes were measured at 48 h post-CuSO_4_ induction or post-transfection with dsRNA, while cell death was assessed at 72 h.

### 2.2. Plasmid Construction

To overexpress *miR-33* in *Drosophila* S2 cells, we constructed the pMT-miR-33 plasmid. A DNA fragment containing the precursor *miR-33* sequence was amplified using Phusion High-Fidelity DNA polymerase (Thermo Fisher Scientific, Waltham, MA, USA) and inserted between the *Xho*I and *Not*I restriction sites of the pMT/V5-His A vector (Thermo Fisher Scientific).

To generate an HA-tagged *Ras64B* (HA-Ras64B) construct containing its 3′-UTR, the *Ras64B* coding sequence and 3′-UTR were amplified using Phusion High-Fidelity DNA polymerase and cloned into the pMT/V5-His A vector (Thermo Fisher Scientific) between the *EcoRI* and *XhoI* restriction sites. Subsequently, an amplified HA DNA fragment was inserted between the *SpeI* and *EcoRI* restriction sites.

For the dual-luciferase reporter plasmid, a DNA fragment containing the wild-type (WT) 3′-UTR of *Ras64B* was amplified using Phusion High-Fidelity DNA polymerase and cloned downstream of the *Renilla* luciferase gene in the psiCHECK-2 vector (Promega, Madison, WI, USA). A mutant (MT) luciferase reporter plasmid containing the 3′-UTR of *Ras64B* with mutations in the predicted miR-33-5p binding sites was generated using a site-directed mutagenesis method, as described previously [21]. All primers used for plasmid construction are provided in Supplementary Table S1.

### 2.3. Determination of miRNA and mRNA Levels

Total RNA was extracted from S2 cells using the TRIzol reagent (Invitrogen, Waltham, MA, USA) according to the manufacturer’s protocol. Subsequently, to eliminate DNA contaminants, the RNA was treated with DNase I (Enzynomics, Daejeon, Republic of Korea). The mature miRNA levels were quantified using a PCR-based miRNA detection method, as described previously, with minor modifications [22,23]. Briefly, after polyadenylation using *E. coli* poly(A) polymerase (Enzynomics), the RNA was reverse-transcribed using M-MLV reverse transcriptase (M-MLV RTase; Enzynomics) and a miR-RT primer containing a poly-dT sequence.

For mRNA level determination, cDNA was synthesized by reverse transcription of RNA using M-MLV RTase and random hexamers (Enzynomics). Quantitative PCR (qPCR) was employed to measure the relative abundances of miRNAs and mRNAs using specific primer sets listed in Supplementary Table S1. qPCR reactions were conducted on a QuantStudio 3 Real-Time PCR instrument (Thermo Fisher Scientific). *U6* snRNA and *rp49* levels served as internal controls for miRNA and mRNA quantification, respectively.

### 2.4. Cell Proliferation and Death

Following pMT-miR-33 or dsRas64B transfection in S2 cells, cell proliferation and death analyses were conducted. Total cell numbers were determined using a hemocytometer under an inverted microscope (Motic, Vancouver, BC, Canada). To assess cell death, cells were incubated with 0.2% Trypan Blue solution (Welgene) in PBS for 5 min, and the number of stained (dead) cells was counted using a hemocytometer under an inverted microscope (Motic). Statistical analyses were performed based on data from four biological replicates.

### 2.5. Gene Ontology (GO) Term Analysis

Gene ontology (GO) enrichment analysis was conducted on genes commonly identified as miR-33-5p targets by both TargetScanFly [24] and PAR-CLIP-seq [25] using the enrichGO function from the R package clusterProfiler (version 4.14.4) [26]. The analysis was performed with the ontology category set to biological process (BP). Among the results, BP terms with high fold enrichment (fold enrichment ≥ 10) and statistical significance (*p* < 0.001) were visualized using the ggplot and geom_point functions from the ggplot2 package (version 3.5.1).

### 2.6. Western Blotting

Western blotting was conducted as previously described with minor modifications [16]. Protein samples from S2 cells were directly prepared in 1× SDS protein loading buffer (58.3 mM Tris-HCl [pH 6.8], 59.3 mM SDS, 7.5% glycerol, 100 mM DTT) and boiled for 10 min. The proteins were separated by SDS–PAGE using 10% polyacrylamide gels and transferred onto nitrocellulose membranes. The primary antibodies used in this study were anti-phosphorylated ERK (1:1000 dilution; Cell Signaling Technology, Danvers, MA, USA), anti-HA (1:1000 dilution; Santa Cruz Biotechnology, Dallas, TX, USA), and anti-b-Tubulin (1:5000 dilution; Developmental Studies Hybridoma Bank, Iowa City, IA, USA). The chemiluminescence signal was detected using a FluorChem HD2 imaging system (proteinsimple, San Jose, CA, USA), and the band intensities were quantified using Image J (version 1.54g) [27].

### 2.7. Dual-Luciferase Reporter Assay

The dual-luciferase reporter assay was performed as previously described [16]. The pMT-miR-33 vector (or pMT empty vector as a control) and the psiCHECK-2 vector, which contains the wild-type (WT) or mutant (MT) *Ras64B* 3′-UTR, were co-transfected into S2 cells using the TransIT-Insect Transfection Reagent. Luciferase activities were quantified using the Dual-Luciferase Reporter Assay System (Promega) according to the manufacturer’s instructions. *Renilla* and firefly luciferase activities were measured using a microplate reader (Molecular Devices, San Jose, CA, USA). Firefly luciferase activity was used for normalization of *Renilla* luciferase activity.

### 2.8. dsRNA Generation

DNA templates were amplified using Phusion High-Fidelity polymerase (Thermo Fisher Scientific) and primer sets containing T7 promoter sequences (Supplementary Table S1). The amplified products were purified using a Zymoclean Gel DNA Recovery Kit (Zymo Research, Irvine, CA, USA). In vitro transcription was performed with T7 RNA polymerase (New England Biolabs, Ipswich, MA, USA), according to the manufacturer’s protocol. DNA templates were subsequently removed by DNase I treatment, and the synthesized RNAs were purified through phenol-chloroform-isoamyl alcohol (PCI; Thermo Fisher Scientific) extraction and ethanol precipitation. The RNAs were annealed in an annealing buffer (10 mM Tris-Cl, pH 8.0 and 20 mM NaCl) by gradually cooling from 85 °C to 25 °C.

### 2.9. Drosophila Melanogaster

All flies were reared on a standard cornmeal, molasses, and yeast medium at 25 °C or 29 °C under non-crowded conditions. The UAS/GAL4 system was employed to overexpress miR-33, miR-33-SP, *Ras64B*, or dsRNA-Ras64B in wing discs. The following transgenic fly lines were obtained from the Bloomington *Drosophila* Stock Center (BDSC) or the Korea *Drosophila* Resource Center (KDRC): *w^1118^* (BDSC #5905), *en2.4*-GAL4 (BDSC #30564), UAS-*Dcr-2*; *en2.4-GAL4*, *UAS-2xEGFP* (BDSC #25752), *en2.4-GAL4*, *UAS-RFP/CyO* (BDSC #30557), *UAS-Ras64B* (BDSC #2025), *UAS-Ras64B-RNAi* (BDSC #29318), *UAS-LUC-miR-33* (BDSC #41150), *UAS-mCherry-miR-33-sponge* (BDSC #61385), *UAS-2xEGFP* (BDSC #6874), and UAS-ERK^D334N^ (KDRC #10106). The *w^1118^* line served as the control strain.

### 2.10. Analysis of Adult Wings

The left wings of adult female flies belonging to the specified genotypes were collected. The wings were subsequently mounted in 50% glycerol in PBS and imaged using a stereomicroscope (Olympus, Tokyo, Japan). The relative dimensions of the posterior wing compartment was quantified using Image J (version 1.54g) [27]. The dimensions of wing cells and their respective counts within the posterior compartment were assessed as previously described [28,29]. Briefly, the number of cells in the posterior compartment was determined by counting the trichomes within a standardized area (50 × 50 pixels), which was consistently positioned across individual wings. The average wing cell size was subsequently calculated by dividing the standardized area by the number of trichomes. The total cell count within the posterior compartment was then estimated by dividing the size of the posterior wing compartment by the average cell size.

### 2.11. Immunostaining of Wing Discs

As previously described [30], wing discs were dissected in cold PBS from wandering third-instar larvae. The specimens were fixed in 4% paraformaldehyde (PFA) for 20 min at 25 °C and subsequently washed three times with PBST (PBS containing 0.2% Triton X-100) for 10 min each. Following washing, the specimens were incubated in blocking solution (10% horse serum in PBS) for 1 h at 25 °C and then incubated overnight at 4 °C with anti-phosphorylated ERK (1:200 dilution; Cell Signaling Technology) in blocking solution. After incubation, the specimens were washed three times with PBST for 10 min each. The samples were then incubated with Alexa Fluor 488 or 594-conjugated secondary antibody (1:500 dilution; Molecular Probes, Eugene, OR, USA) for 1 h at 25 °C. Finally, the specimens were mounted in mounting medium (Abcam, Cambridge, UK) and imaged using a confocal laser-scanning microscope (CarlZeiss, Oberkochen, Germany).

### 2.12. Analysis of Wing Disc Size

Wing discs were dissected in cold PBS from wandering third-instar larvae of the specified genotypes. The specimens were fixed in 4% PFA for 20 min at 25 °C and subsequently washed three times with PBST for 10 min each. The samples were mounted in a mounting medium containing 4′,6-diamidino-2-phenylindole (DAPI) (Abcam). Fluorescence images were captured using a Zeiss Axio Imager M1 fluorescence microscope (Carl Zeiss). The size of posterior compartment, marked by RFP, was determined using Image J-win64 software [27].

## 3. Results

### 3.1. miR-33 Negatively Regulates Cell Growth in Drosophila S2 Cells

**Figure 1:**
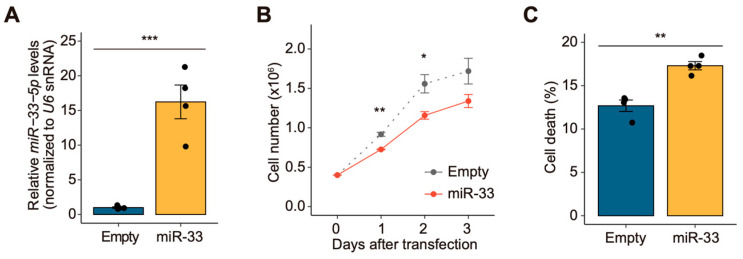
*miR-33* overexpression inhibits cell growth in *Drosophila* S2 cells. (**A**) Overexpression of *miR-33-5p* in S2 cells (*n* = 4). The empty vector (Empty) served as a control, and *U6 snRNA* levels were used as an internal control to normalize miRNA expression levels. (**B**) Cell proliferation in *miR-33*-overexpressing S2 cells (*n* = 3). (**C**) Percentage of dead cells in *miR-33*-overexpressing S2 cells 72 h post-transfection (*n* = 4). All data are presented as the mean ± standard error of the mean (SEM). Statistical significance was determined using Student’s *t*-test: * *p* < 0.05, ** *p* < 0.01, and *** *p* < 0.001 compared to the control.

Previous studies have demonstrated that miR-33 regulates triacylglyceride synthesis in *Drosophila* by targeting the *atpcl*, *midway*, and *Akt1* genes [20]. In this study, we sought to explore the additional biological functions of miR-33 (accession number: MI0000364) in *Drosophila* and investigate its potential cellular roles. To achieve this, we first generated a *miR-33* expression construct and confirmed the overexpression of *miR-33-5p* (accession number: MIMAT0000342), the primary strand of miR-33, in *Drosophila* S2 cells (Figure 1A). Subsequently, under conditions of *miR-33* overexpression, we analyzed cellular phenotypic changes. Notably, cell proliferation was significantly reduced in *miR-33*-overexpressing S2 cells compared to control cells (Figure 1B). Additionally, the cell death rate was moderately increased in *miR-33*-overexpressing S2 cells 72 h post-transfection (Figure 1C). These observations collectively indicate that miR-33 plays a role in the negative regulation of cell growth in *Drosophila* S2 cells.

### 3.2. miR-33-5p Suppresses Ras64B in Drosophila

**Figure 2:**
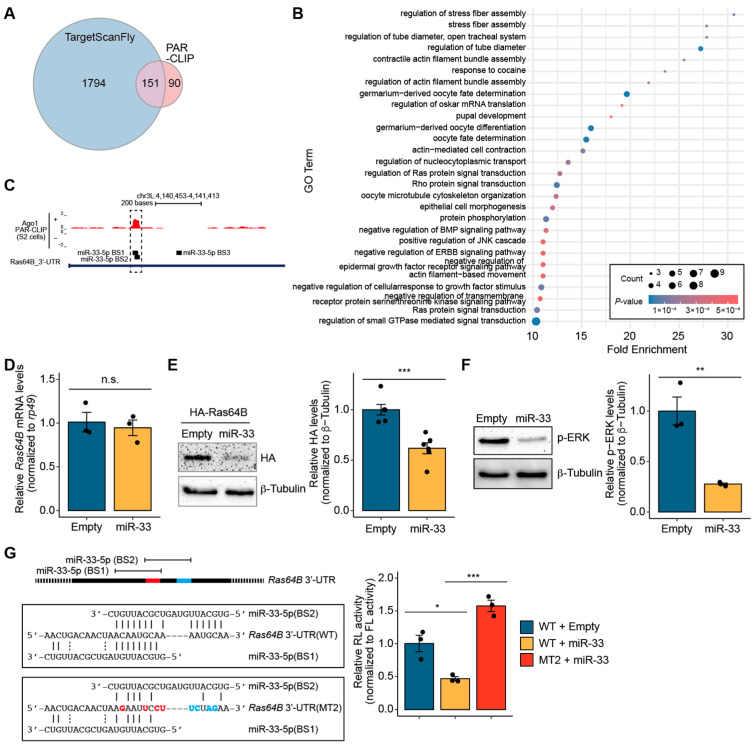
miR-33-5p targets *Ras64B* in *Drosophila*. (**A**) Venn diagram illustrating the overlap of potential miR-33-5p targets analyzed from the TargetScanFly and Ago1 PAR-CLIP-seq data. (**B**) Functional enrichment analysis of the common miR-33-5p targets predicted using TargetScanFly and Ago1 PAR-CLIP-seq. Significant Gene Ontology (GO) biological processes that were overrepresented are shown (*p* < 0.001 and fold enrichment ≥ 10). (**C**) Ago1 PAR-CLIP-seq signals (red) and predicted miR-33-5p binding sites (BS; black lines) in the *Ras64B* 3′-UTR in S2 cells. The overlapping region of Ago1 PAR-CLIP-seq signals and miR-33-5p BS is marked with a dotted box. (**D**) Relative levels of *Ras64B* mRNA transcripts in *miR-33*-overexpressing S2 cells (*n* = 3). The expression levels of *rp49* were used as an internal control. (**E**) Relative HA-Ras64B protein levels under *miR-33* overexpression. Representative Western blot (**left**) and quantification data (**right**; *n* = 6) are shown. b-Tubulin served as a loading control. (**F**) p-ERK protein levels in *miR-33*-overexpressing S2 cells. The normalized p-ERK values relative to b-Tubulin are presented. Representative Western blot (**left**) and quantification data (**right**; *n* = 3) are shown. (**G**) Luciferase reporter assay in S2 cells. The sequences of miR-33-5p and its predicted binding site at the wild-type (WT) or mutant (MT2) 3′-UTR of *Ras64B* are depicted (**left**). Mutated sequences are indicated in red (the seed region of BS1) or blue (the seed region of BS2). *Renilla* luciferase (RL) activity is presented as a bar plot and normalized to the firefly luciferase (FL) activity (**right**) (*n* = 3). All bar plots represent the mean ± SEM. Statistical significance was assessed using Student’s *t*-test (**D**–**F**) or analysis of variance (ANOVA) with a supplementary Dunnett’s test (**G**): n.s., not significant; * *p* < 0.05, ** *p* < 0.01, and *** *p* < 0.001 compared to the control.

Given the association between miR-33 and cell growth, we next sought to uncover the molecular mechanisms regulated by miR-33 in *Drosophila*. To identify potential target genes of the primary strand miR-33-5p, we analyzed predicted targets using both the TargetScanFly database and Ago1 PAR-CLIP-seq data from S2 cells [24,25]. TargetScanFly predicted a total of 1945 conserved and non-conserved target genes, while PAR-CLIP-seq analysis identified 241 potential target genes (Figure 2A and Supplementary Table S2). Among these, 151 genes were commonly identified in both datasets (Figure 2A and Supplementary Table S2). Using these 151 overlapping predicted target genes, gene ontology (GO) term analysis was performed, revealing 28 significantly enriched biological processes (*p* < 0.001 and fold enrichment ≥ 10) (Figure 2B). Notably, several biological processes closely associated with cell growth were identified, including “Negative regulation of epidermal growth factor receptor signaling pathway” and “Ras protein signal transduction”. Within the “Ras protein signal transduction” category, there are multiple predicted target genes of miR-33-5p, including *Ras64B*, Son of sevenless (*Sos*), β subunit of type I geranylgeranyl transferase (*βggt-I*), daughter of sevenless (*dos*), and sprouty (*sty*). Based on our findings, PAR-CLIP-seq signals and previous literature, we selected *Ras64B* for further experimental validation.

In *Drosophila* Ago1 PAR-CLIP-seq data [25], we identified a prominent Ago1 binding peak within the 3′-UTR of *Ras64B* (Figure 2C). Subsequent analysis utilizing the TargetScanFly database [24] revealed three potential miR-33-5p binding sites (BS1, BS2, and BS3), with two overlapping sites (BS1 and BS2) located within the Ago1 binding peak region (Figure 2C). These findings suggest that miR-33-5p may directly target *Ras64B* to regulate its expression.

To ascertain whether miR-33 regulates *Ras64B* expression, we initially examined the transcript levels of *Ras64B* in *miR-33*-overexpressing S2 cells. However, *Ras64B* mRNA levels remained unchanged under *miR-33* overexpression conditions relative to the control (Figure 2D). Moreover, the expression levels of other genes belonging to the “Ras protein signaling transduction” category were also unchanged in *miR-33*-overexpressing S2 cells (Supplementary Figure S1A–D).

Given the absence of alterations at the RNA level, we next investigated the relationship between miR-33 and *Ras64B* employing alternative approaches. Because specific antibodies against *Drosophila* Ras64B protein were unavailable, we utilized an HA-tagged Ras64B (HA-Ras64B) construct containing its 3′-UTR. Notably, HA-Ras64B protein levels were reduced under *miR-33* overexpression compared with the control (Figure 2E and File S1). Furthermore, we analyzed the activity of the known pathway in which Ras64B is involved [31]. Specifically, we measured the levels of phosphorylated ERK (p-ERK) protein, a downstream factor of the Ras signaling pathway, in *miR-33*-overexpressing S2 cells. As expected, p-ERK protein levels were significantly reduced in S2 cells overexpressing *miR-33* compared to controls (Figure 2F and File S1).

To determine whether miR-33 directly binds to the predicted target sites within the *Ras64B* 3′-UTR, we performed a dual-luciferase reporter assay using constructs containing either the wild-type (WT) or mutant (MT) *Ras64B* 3′-UTR. Initially, we generated a mutant *Ras64B* 3′-UTR construct (Ras64B MT1 3′-UTR), wherein a segment of the sequence overlapping both BS1 and BS2, including the seed region of BS1, was mutated (Supplementary Figure S1E). Subsequently, we overexpressed *miR-33*, which significantly reduced the activity of the *Renilla* luciferase reporter harboring the *Ras64B* WT 3′-UTR (Supplementary Figure S1E). However, contrary to our anticipations, miR-33-mediated suppression of *Renilla* luciferase activity was only marginally attenuated in S2 cells expressing the reporter with the *Ras64B* MT1 3′-UTR (Supplementary Figure S1E). To further elucidate the contribution of BS2, we additionally mutated the seed region of BS2 (Ras64B MT2 3′-UTR) and repeated the experiment. Remarkably, the miR-33-mediated suppression effect was profoundly abolished in S2 cells expressing the *Renilla* luciferase reporter containing the *Ras64B* MT2 3′-UTR (Figure 2G). In addition, consistent with the unchanged *Ras64B* mRNA levels observed upon *miR-33* overexpression, the mRNA levels of the *Renilla* luciferase reporter were not affected by *miR-33* overexpression (Supplementary Figure S1F).

Taken together, these findings demonstrate that miR-33-5p directly targets the 3′-UTR of *Ras64B* to negatively regulate its expression at the translational level. Furthermore, while both BS1 and BS2 contribute to this regulation, the suppression effect appears to be more dependent on BS2 binding.

### 3.3. Ras64B Is Involved in Cell Proliferation in S2 Cells

**Figure 3:**
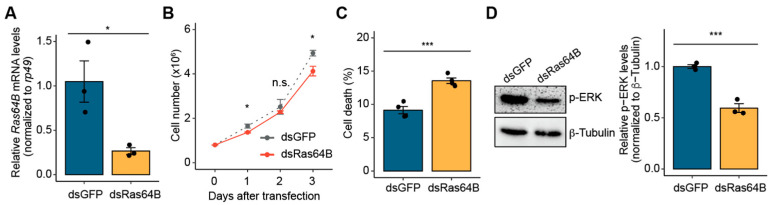
*Ras64B* depletion reduces cell proliferation in *Drosophila* S2 cells. (**A**) Relative expression levels of *Ras64B* mRNA (*n* = 3): dsRas64B treatment reduced the relative expression levels of *Ras64B* mRNA in S2 cells compared to dsGFP treatment, which served as a control. (**B**) Cell proliferation (*n* = 4): dsRas64B treatment significantly decreased cell proliferation in S2 cells. (**C**) Percentage of dead cells (*n* = 4): 72 h after dsRas64B treatment, the percentage of dead cells in S2 cells was significantly higher compared to the control. (**D**) p-ERK protein levels: dsRas64B treatment reduced the p-ERK protein levels in S2 cells relative to b-Tubulin, which served as a loading control. Representative Western blot (**left**) and quantification data (**right**; *n* = 3) are shown. All bar plots represent the mean ± SEM. Statistical significance was determined using Student’s *t*-test: n.s., not significant; * *p* < 0.05 and *** *p* < 0.001 compared to the control.

Given that miR-33-5p suppresses *Ras64B* in S2 cells, we next sought to determine whether *Ras64B* is directly linked to cell proliferation in these cells. To knock down *Ras64B*, double-stranded RNAs against *Ras64B* mRNA (dsRas64B) were generated using an in vitro transcription method. The knockdown efficiency (Figure 3A) and specificity (no targeting of *Ras85D*; Supplementary Figure S2) of dsRas64B were confirmed.

Under *Ras64B* depletion conditions, S2 cells exhibited a significant reduction in cell proliferation compared to dsGFP-treated control cells (Figure 3B), consistent with the observations from *miR-33*-overexpressing S2 cells. Additionally, dsRas64B-treated S2 cells showed increased cell death relative to controls (Figure 3C). These findings indicate that *Ras64B* plays a role in regulating cell growth in S2 cells. Given that *Ras64B* is a direct target of miR-33-5p, these results suggest that miR-33-mediated regulation of cell growth is linked to *Ras64B* expression.

To further explore the role of *Ras64B* in the ERK signaling pathway, we investigated whether *Ras64B* depletion affects p-ERK levels in S2 cells. Consistent with the results of *miR-33* overexpression, p-ERK levels were reduced in dsRas64B-treated S2 cells compared to controls (Figure 3D and File S1). This observation supports the notion that *Ras64B* is involved in the ERK signaling pathway associated with cell growth.

### 3.4. miR-33 Regulates Drosophila Wing Size

**Figure 4:**
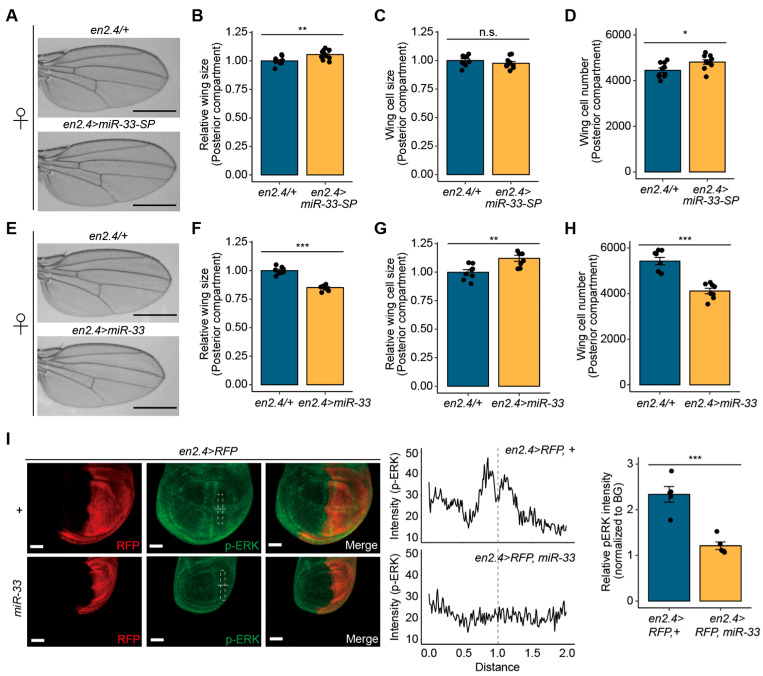
miR-33 regulates wing size in *Drosophila*. (**A**) Representative images of wings from the *en2.4 > miR-33-sponge (SP)* female flies. The *en2.4/+* female flies served as the control group. Scale bar: 0.5 mm. (**B**) Posterior wing compartment size in *en2.4 > miR-33-SP* female flies (*n* = 10). (**C**) Posterior wing compartment cell size in *en2.4 > miR-33-SP* female flies (*n* = 10). (**D**) The total number of cells in the posterior wing compartment of *en2.4 > miR-33-SP* female flies (*n* = 10). (**E**) Representative wing image of *en2.4 > miR-33* female flies. (**F**) Posterior wing compartment size in *en2.4 > miR-33* female flies (*n* = 8). (**G**) Posterior wing compartment cell size in *en2.4 > miR-33* female flies (*n* = 8). (**H**) The total number of cells in the posterior wing compartment of *en2.4 > miR-33* female flies (*n* = 8). (**I**) p-ERK expression in the wing discs of *en2.4 > RFP*, *miR-33* larvae (**left**). RFP (posterior compartment) and p-ERK are marked in red and green, respectively. The p-ERK intensity profiles in the wing discs of *en2.4 > RFP*, *miR-33* larvae (**middle**) are outlined by dotted boxes. The central line in the intensity plot indicates the D/V boundary. The relative p-ERK intensity (*n* = 5) was normalized to background intensity (BG; **right**). Scale bars, 50 μm. All bar plots show mean ± SEM. Statistical significance was determined using Student’s *t*-test: n.s., not significant; * *p* < 0.05, ** *p* < 0.01, and *** *p* < 0.001 compared to the control.

To extend our findings from S2 cells to the in vivo biological function of miR-33, we selected the *Drosophila* wing as a well-established tissue model for cell growth analysis. To this end, *miR-33* was depleted in the posterior compartment of wings by crossing validated miRNA sponge (SP) flies targeting miR-33 [20] with *en2.4-GAL4* flies (*en2.4* > *miR-33-SP*). The posterior compartment size of wings was significantly increased in *en2.4* > *miR-33-SP* flies compared with the controls (Figure 4A,B). Along with this, the anterior/posterior (A/P) area ratio was reduced in *en2.4* > *miR-33-SP* wings relative to the controls (Supplementary Figure S3A). We further examined whether the expansion of the posterior compartments resulted from changes in cell size, cell number, or both. Analysis of the wing posterior compartment revealed no significant changes in cell size between *en2.4* > *miR-33-SP* and *en2.4*/*+* control flies (Figure 4C). However, cell number was significantly increased in *en2.4* > *miR-33-SP* flies (Figure 4D), suggesting that miR-33 depletion promotes cell proliferation.

To further investigate the roles of miR-33 in wings, we overexpressed *miR-33* in the posterior compartment of wings by crossing between *UAS-miR-33* flies with *en2.4-GAL4* flies (*en2.4* > *miR-33*). The posterior compartment size was significantly reduced in the wings of *en2.4* > *miR-33* adult flies compared to those of *en2.4*/*+* control flies (Figure 4E,F). Consistently, the A/P area ratio was increased in the *en2.4* > *miR-33* wings relative to the controls (Supplementary Figure S3B). In addition, the anterior cross vein (ACV) was either absent or weakened in the wings of *en2.4* > *miR-33* adult flies (Figure 4E).

Further analysis of cell size and number in the wings of *en2.4 > miR-33* flies revealed a slight increase in cell size (Figure 4G), but a significant decrease in cell number compared to controls (Figure 4H). These observations suggest that miR-33-mediated wing growth defects result from a reduction in cell number rather than cell size. Taken together, consistent with the observations from S2 cells, these findings demonstrate that miR-33 plays a crucial role in regulating growth during *Drosophila* wing development by modulating cell number.

Furthermore, we hypothesized a link between miR-33 and ERK signaling in wing discs, as observed in S2 cell. To assess p-ERK levels, we performed immunofluorescence staining for p-ERK in wing discs. In control wing discs of *en2.4* > *RFP*, +larvae, two distinct stripes of p-ERK signals were clearly detected running parallel to the dorsal/ventral (D/V) and A/P boundaries of the wing pouch. Conversely, in wing discs of *en2.4* > *RFP*, *miR-33* larvae, p-ERK signals exhibited a marked reduction (Figure 4I). These findings substantiate miR-33’s involvement in the ERK signaling pathway, further reinforcing its role in growth regulation.

### 3.5. miR-33-Mediated Wing Growth Defects in Drosophila Are Linked to Ras64B

**Figure 5:**
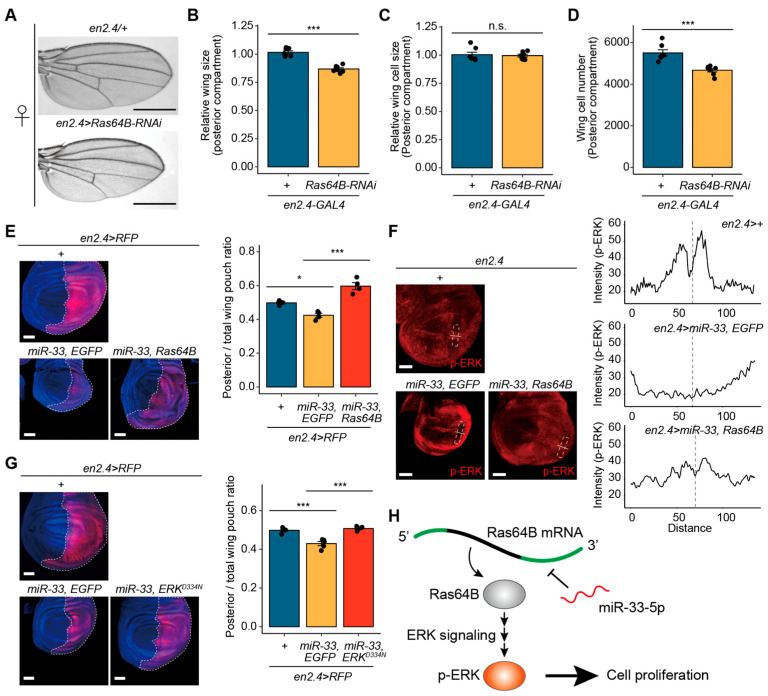
miR-33-mediated wing defects are associated with *Ras64B* in *Drosophila*. (**A**) Representative images of wings from *en2.4 > Ras64B-RNAi* female flies. *en2.4/+* female flies served as controls. Scale bar, 0.5 μm. (**B**) Relative size of the posterior wing compartment in *en2.4 > Ras64B-RNAi* flies (females, *n* = 7). (**C**) Relative cell size in the posterior wing compartment of *en2.4 > Ras64B-RNAi* flies (females, *n* = 7). (**D**) Total cell number in the posterior wing compartment of *en2.4 > Ras64B-RNAi* flies (females, *n* = 7). (**E**) Increase in the posterior wing compartment of *en2.4 > miR-33*, *Ras64B* larvae. Representative images of wing discs from *en2.4 > miR-33*, *Ras64B* larvae (**left**). The posterior compartment is outlined with a dotted line. Quantitative data of the posterior-to-total wing pouch area ratio are shown in the bar plot (*n* = 4, **right**). (**F**) p-ERK expression in the posterior compartment of *en2.4 > miR-33*, *Ras64B* larval wing discs (**left**). p-ERK is marked in red. The p-ERK intensity profiles are shown (**right**; marked by dotted boxes in the wing discs). The central line in the intensity plot indicates the D/V boundary. (**G**) Increase in the posterior wing compartment of *en2.4 > miR-33*, *ERK^D334N^* larvae. Representative images of wing discs from *en2.4 > miR-33*, *ERK^D334N^* larvae (**left**). Quantitative data of posterior-to-total wing pouch area ratio are shown in the bar plot (*n* = 5, **right**). Scale bars, 50 μm. (**H**) Schematic diagram illustrating the miR-33-*Ras64B*-ERK regulatory axis. In all wing disc images, the anterior compartment is positioned on the left and the posterior compartment on the right. All bar plots represent mean ± SEM. Statistical significance was determined using Student’s *t*-test (**B**–**D**) or ANOVA with a supplementary Dunnett’s test (**E**,**G**): n.s., not significant; * *p* < 0.05 and *** *p* < 0.001 compared to the control.

To investigate whether the reduction in wing growth mediated by *miR-33* overexpression is associated with *Ras64B*, we initially examined the phenotypic changes in the wings of *en2.4* > *Ras64B-RNAi* flies (*Ras64B* depleted in the wing posterior compartment). Consistent with the observations from *miR-33* overexpression, the posterior wing compartment’s size was significantly reduced in *en2.4* > *Ras64B-RNAi* flies compared to controls (Figure 5A,B). In addition, the A/P area ratio was increased in *en2.4* > *Ras64B-RNAi* wings relative to the controls (Supplementary Figure S3C). Furthermore, defects in ACV were observed in the wings of *en2.4* > *Ras64B-RNAi* flies (Figure 5A).

To determine whether the diminution of the posterior compartment was attributed to alterations in cell size, cell number, or both, we conducted further analyses. The results indicated no significant changes in cell size in the posterior compartment (Figure 5C), whereas the cell number in the posterior compartment was decreased in *en2.4* > *Ras64B-RNAi* flies compared to controls (Figure 5D). These findings suggest that *Ras64B* is implicated in wing growth by regulating cell number.

Furthermore, we examined whether the wing growth defects induced by *miR-33* overexpression in the posterior wing compartment could be rescued by *Ras64B* co-overexpression. However, when miR-33 and *Ras64B* were co-expressed in the wing posterior compartment using *en2.4-GAL4*, *en2.4* > *miR-33*, *Ras64B* larvae exhibited lethality prior to reaching the pupal stage. Consequently, we were unable to analyze wings of adult flies. Nevertheless, analysis of larval wing discs revealed a significant increase in the posterior-to-total wing pouch area ratio in *en2.4 > miR-33*, *Ras64B* larval wing discs compared with controls (Figure 5E), supporting the hypothesis that miR-33-induced wing growth defect is mediated through *Ras64B*. As expected, the p-ERK signal was broadly increased throughout the posterior compartment in *en2.4 > miR-33*, *Ras64B* larval wing discs compared with the anterior compartment, and the characteristic p-ERK stripe pattern was partially restored in these discs (Supplementary Figure S4 and Figure 5F). Additionally, we examined whether the reduction in wing disc size induced by *miR-33* overexpression could be rescued by co-expressing a constitutively active form of *Drosophila* ERK (ERK^D334N^) [32]. Indeed, the posterior-to-total wing pouch area ratio in the larval wing discs of *en2.4 > miR-33*, *ERK^D334N^* larvae was significantly increased compared with that of the *en2.4 > miR-33*, *EGFP* control (Figure 5G). Collectively, these findings demonstrate that miR-33-5p regulates cell growth by targeting *Ras64B*, which is involved in the ERK signaling pathway in *Drosophila* (Figure 5H).

## 4. Discussion

In this study, our findings demonstrate that the interaction between miR-33-5p and ERK signaling occurs not only in *Drosophila* S2 cells but also during wing development. Overexpression of *miR-33* in the posterior compartment of the wing resulted in a reduction in posterior size, accompanied by a decrease in cell number and a reduction in p-ERK levels (Figure 4). These miR-33-mediated phenotypic changes in the wing are likely attributed to the downregulation of its target gene, *Ras64B*. To further assess the functional interaction between miR-33-5p and *Ras64B* in wing growth, we attempted to determine whether co-expression of *Ras64B* could rescue the miR-33-5p-induced phenotypic changes by measuring the posterior compartment size and cell number. However, when we used *en2.4-GAL4*, which induces gene expression specifically in the wing posterior compartment, to express *Ras64B* alone or together with miR-33, we observed lethality at the larval stage. This larval lethality may reflect excessive activation of the RAS/ERK pathway. Consequently, adult wings could not be analyzed. Instead, we confirmed the interaction between miR-33-5p and *Ras64B* in regulating wing growth by analyzing larval wing discs (Figure 5E,F). Future studies employing temporally controlled or weaker GAL4 drivers, or examining other tissues, will further clarify the interaction between these two molecules in the regulation of *Drosophila* growth.

Our findings also indicate that either *miR-33* overexpression or *Ras64B* depletion results in diminished cell growth by inhibiting cell proliferation. In *Drosophila*, the Ras/ERK signaling pathway is closely associated with cell proliferation. For instance, the AP-1 transcription factor Fos, a downstream effector of ERK, promotes intestinal stem cell (ISC) proliferation [33]. Activated ERK signaling phosphorylates the HMG-box transcriptional repressor Capicua (Cic), leading to its cytoplasmic translocation. This, in turn, facilitates ISC proliferation by inducing the transcription of *stg*, *CycE*, *Ets21C*, and *pnt* [34]. Furthermore, Ras signaling upregulates *dMyc*, which promotes cell proliferation by elevating CycE levels, thereby facilitating G1/S progression [8]. In addition, we observed elevated cell death upon *miR-33* overexpression and *Ras64B* depletion. Previous reports have shown that Ras pathway activation suppresses *rpr*- or *hid*-induced apoptosis in the developing eye [35]. In embryos, constitutively active Ras downregulates *hid* mRNA expression, whereas an active form of *yan*, a negative regulator of the Ras/MAPK pathway, induces cell death [36]. These pathways may therefore contribute to the reduced cell proliferation mediated by *miR-33* overexpression or *Ras64B* depletion.

Using a combination of target prediction program and Ago1 PAR-CLIP-seq data, we selected potential targets of miR-33-5p. Among them, several genes—*Ras64B*, *Sos*, *bggt-I*, *dos*, and *sty*—were classified under the “Ras protein signal transduction” category. The PAR-CLIP-seq data indicated that the miR-33 binding sites within the 3′-UTRs of *Sos* and *bggt-I* exhibited much weaker Ago1 binding signals compared with those of *Ras64B*, *dos*, and *sty*, implying that miR-33-5p is more likely to be functionally associated with *Ras64B*, *dos*, and *sty* in the S2 cell context. *Dos* functions as an adaptor protein linking *sevenless* and *Ras* in *Drosophila*, and dos mutants showed reduced wing size and a loss of the L4 vein [37]. In contrast, *sty* overexpression has been reported to suppress p-ERK signaling [38]. We found that the mRNA levels of all four genes were not significantly changed under *miR-33* overexpression, similar to *Ras64B*. However, we cannot completely rule out the involvement of these genes, given the possibility of miRNA-mediated translational suppression. Furthermore, a subset of the remaining ~150 predicted targets may also contribute to miR-33-mediated regulation of cell growth. These potential interactions could account for the subtle differences observed between *miR-33* overexpression and *Ras64B* depletion, including reduced cell growth, increased cell death, and the enlarged wing cell size of *en2.4* > *miR-33* flies. Future studies will further investigate this complex regulatory network associated with miR-33.

Notably, *Drosophila* wings overexpressing *miR-33* or *Ras64B-RNAi* driven by *en2.4-GAL4* exhibited a loss of ACV (Figure 4E and Figure 5A). Previous studies have demonstrated that ACV formation is associated with multiple signaling pathways, including bone morphogenetic protein (BMP) signaling. For example, deletion of crossveinless (*cv*), which activates BMP signaling, results in ACV loss [39]. These observations suggest that aberrant regulation of ERK signaling by miR-33 and *Ras64B* may be linked to BMP signaling activity. Alternatively, other miR-33 targets could contribute to ACV loss by controlling BMP signaling. Further research is required to elucidate the relationship between miR-33 and BMP signaling.

Given that miR-33 is a highly conserved miRNA from *Drosophila* to humans, it is plausible that its regulation of Ras/ERK signaling is conserved across species. Indeed, a miRNA target prediction database, TargetScan, has predicted K-Ras and N-Ras as potential targets of miR-33 in mice. Furthermore, since K-Ras has been reported to preferentially activate Raf-1 as a downstream effector [6], it is conceivable that the interaction between miR-33 and *Ras64B* is conserved in mice. In humans, a study has demonstrated that miR-33 downregulates p-ERK and p-AKT levels by targeting key enzymes involved in fatty acid oxidation [40]. Taken together with our results, these findings support a conserved role for miR-33 in regulating Ras/ERK signaling across species. Collectively, our study provides novel insights into the function of miRNAs in regulating cell growth.

## 5. Conclusions

This study revealed that miR-33-5p regulates cell proliferation in *Drosophila* by suppressing *Ras64B*. *miR-33* overexpression in S2 cells led to a significant reduction in proliferation accompanied by increased cell death. These cellular phenotypes were linked to *Ras64B*-mediated inactivation of ERK signaling. Consistently, in wings, overexpression of miR-33 reduced cell proliferation and ERK activity, supporting a biological role for the miR-33–*Ras64B*–ERK axis in growth regulation. Collectively, these findings highlight a novel mechanism by which miR-33-5p controls cell and tissue growth through repression of ERK signaling.

## Data Availability

The original contributions presented in this study are included in the article/Supplementary Materials. Further inquiries can be directed to the corresponding authors.

## Supplementary Materials

The following supporting information can be downloaded at https://www.mdpi.com/article/10.3390/biology14121693/s1, Supplementary Figure S1: Regulation of target genes by miR-33.; Supplementary Figure S2: Specificity of dsRas64B targeting *Ras64B* mRNA, not *Ras85D*.; Supplementary Figure S3: Anterior-to-posterior compartment area ratio in wings of adult flies.; Supplementary Figure S4: Relative p-ERK levels in the posterior compartment of *en2.4 > miR-33*, *Ras64B* larval wing discs; Supplementary Table S1: Sequences of the oligonucleotides used in this study.; Supplementary Table S2: Potential target genes for miR-33-5p obtained from TargetScanFly and PAR-CLIP-seq. File S1: original images of WB.
