# Supplementary material for: Drosophila miR-33-5p Suppresses Cell Growth by Inhibiting ERK Signaling
Source: Biology (Basel). 2025 Nov 28;14(12):1693. doi: 10.3390/biology14121693 (PMC12730946; doi:10.3390/biology14121693)
Supplement: Supplementary file 1 [file biology-14-01693-s001.zip › Supplementary_Figure_S1.pdf]

## Supplementary Figure S1

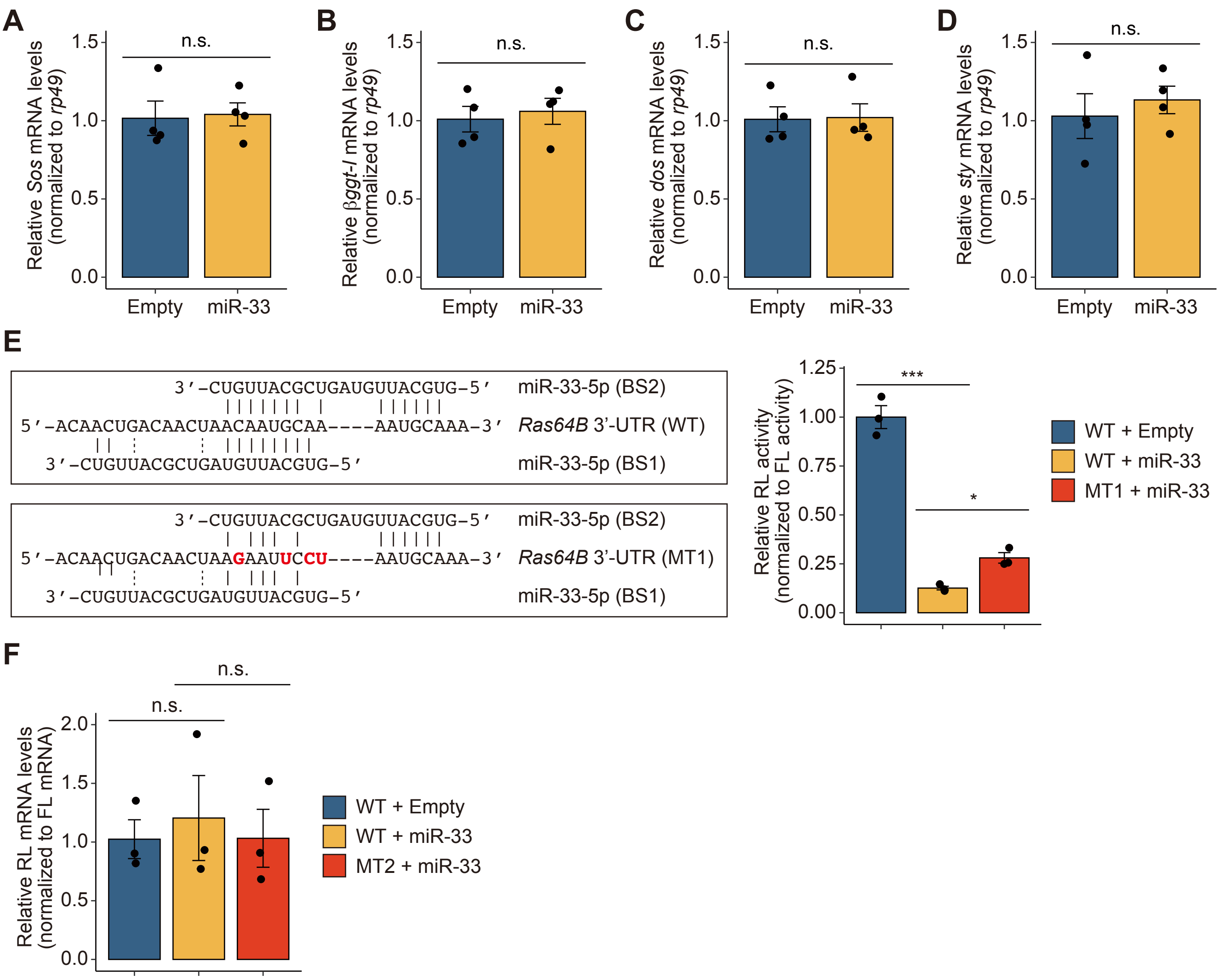


**Supplementary Figure S1. Regulation of target genes by miR-33.** (A, B, C, D) Relative levels of *Sos*, β*ggt-I*, *dos*, and *sty* mRNA transcripts in *miR-33*-overexpressing S2 cells. (E) Luciferase reporter assay for miR-33-5p targeting on the 3′-UTR of *Ras64B* in S2 cells. The sequences of miR-33-5p and its predicted binding site at the wild-type (WT) or mutant (MT1) 3′-UTR of *Ras64B* are shown (left). Mutated sequences are indicated in red. Renilla luciferase (RL) activity is shown as a bar plot and normalized to the firefly luciferase (FL) activity (right). (F) Relative RL mRNA levels of the luciferase reporter contatining the 3′-UTR of *Ras64B*. RL mRNA levels was normalized to FL mRNA levels. Bar plots represent the mean ± SEM. Statistical significance was assessed using Student's *t*-test (A, B, C, D, F) or analysis of variance (ANOVA) with a supplementary Dunnett's test (E): n.s., not significant; *$P < 0.05$ and ***$P < 0.001$ compared with the control.
