# Supplementary material for: Drosophila miR-33-5p Suppresses Cell Growth by Inhibiting ERK Signaling
Source: Biology (Basel). 2025 Nov 28;14(12):1693. doi: 10.3390/biology14121693 (PMC12730946; doi:10.3390/biology14121693)
Supplement: Supplementary file 1 [file biology-14-01693-s001.zip › Supplementary_Figure_S2.pdf]

Supplementary Figure S2

A

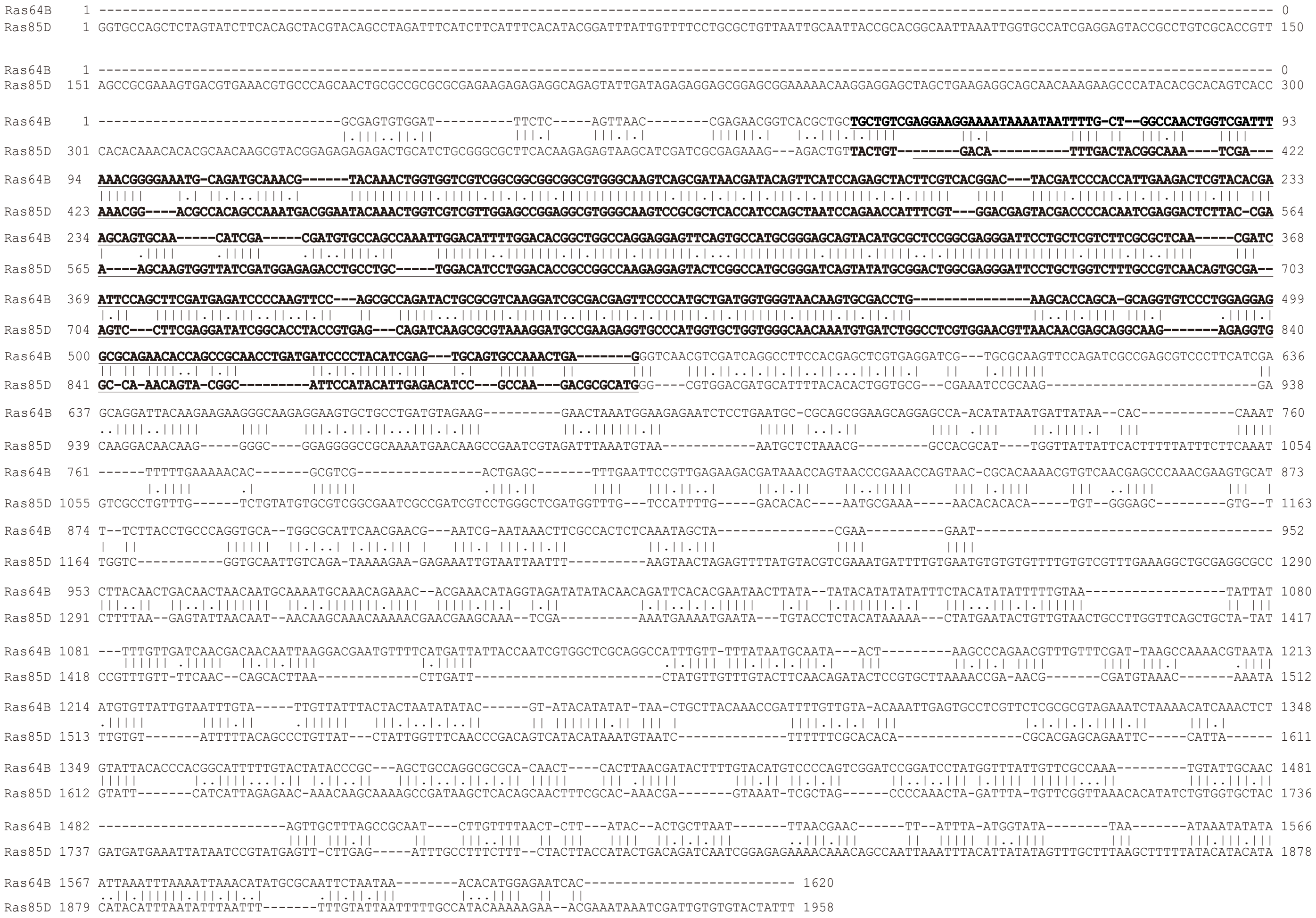

B

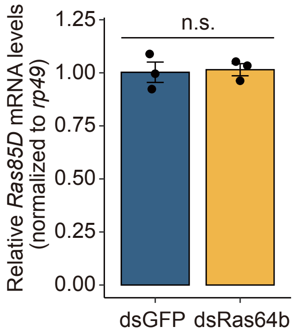

Supplementary Figure S2. Specificity of dsRas64B targting *Ras64B* mRNA, not *Ras85D*. (A) Alignment of *Ras64B* and *Ras85D* mRNA sequences. The sequneces targeted by dsRas64B are highlighted in bold and underlined. (B) Relative expression levels of *Ras85D* mRNA in dsRas64B-treated S2 cells. dsGFP treatment was used as a control. Bar plots represent as the mean ± SEM. Statistical significance was determined using Student's *t*-test: n.s., not significant, compared with control.
