# Supplementary material for: Drosophila miR-33-5p Suppresses Cell Growth by Inhibiting ERK Signaling
Source: Biology (Basel). 2025 Nov 28;14(12):1693. doi: 10.3390/biology14121693 (PMC12730946; doi:10.3390/biology14121693)
Supplement: Supplementary file 1 [file biology-14-01693-s001.zip › Supplementary_Figure_S3.pdf]

Supplementary Figure S3

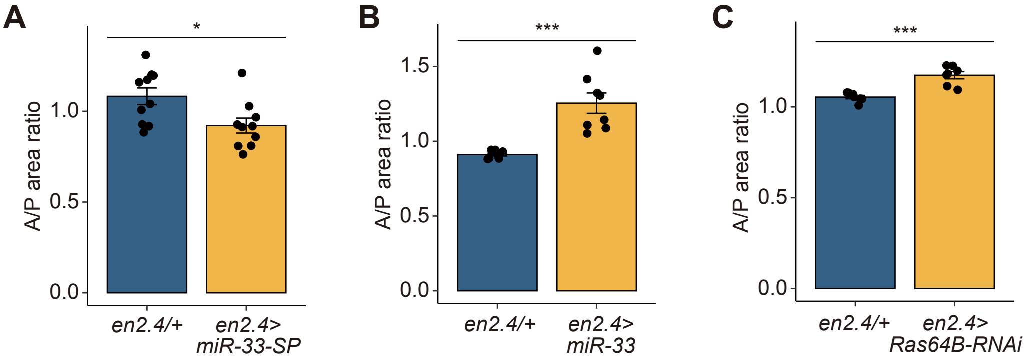

Supplementary Figure S3. Anterior-to-postrior compartment area ratio in wings of adult flies. (A) Anterior-to-posterior (A/P) area ratio in *en2.4 > miR-33-SP* female flies (*n* = 10) (B) A/P area ratio in *en2.4 > miR-33* female flies (*n* = 8). (C) A/P area ratio in *en2.4 > Ras64B-RNAi* female flies (*n* = 7). Bar plots represent as the mean ± SEM. Statistical significance was determined using Student's *t*-test: *$P < 0.05$ and ***$P < 0.001$, compared with the control.
