# Supplementary material for: Drosophila miR-33-5p Suppresses Cell Growth by Inhibiting ERK Signaling
Source: Biology (Basel). 2025 Nov 28;14(12):1693. doi: 10.3390/biology14121693 (PMC12730946; doi:10.3390/biology14121693)
Supplement: Supplementary file 1 [file biology-14-01693-s001.zip › Supplementary_Figure_S4.pdf]

Supplementary Figure S4

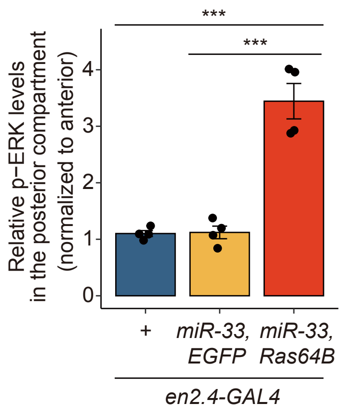

Supplementary Figure S4. Relative p-ERK levels in the posterior compartment of *en2.4 > miR-33, Ras64B* larval wing discs. The p-ERK signal intensity in the posterior compartment was normalized to that of the anterior compartment ($n = 4$). Bar plots represent as the mean ± SEM. Statistical significance was determined using ANOVA with a supplementary Dunnett's test: ***$P < 0.001$, compared with the control.
