# Supplementary figures and images for: Drosophila miR-33-5p Suppresses Cell Growth by Inhibiting ERK Signaling
Source: Biology (Basel). 2025 Nov 28;14(12):1693. doi: 10.3390/biology14121693 (PMC12730946; doi:10.3390/biology14121693)

Figure 2E

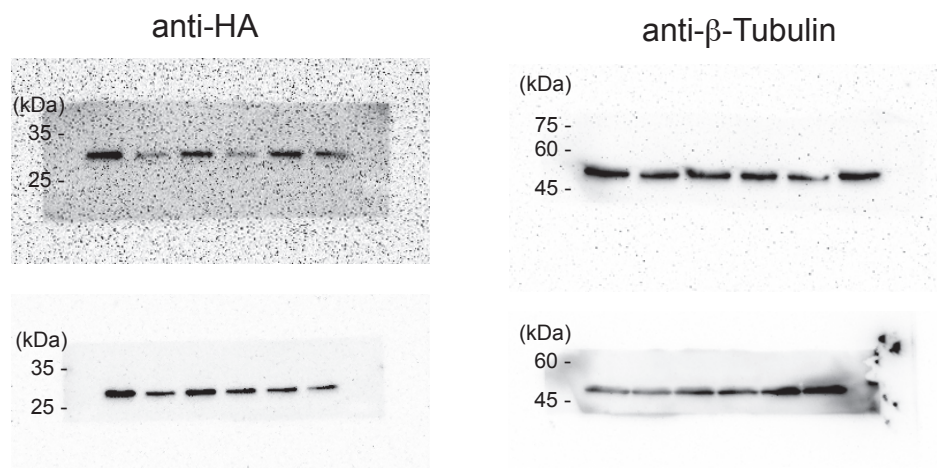

Figure 2F

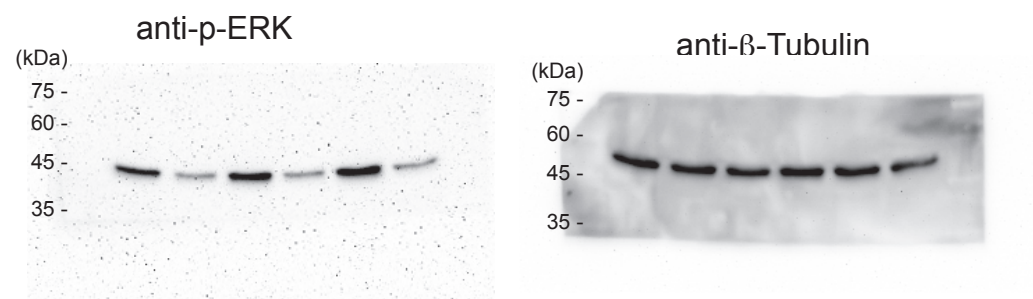

Figure 3D

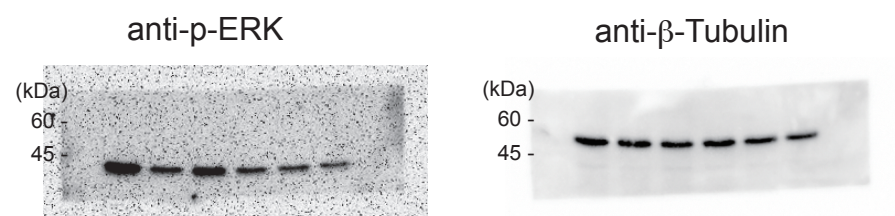

Supplement: Supplementary file 1 [file biology-14-01693-s001.zip › File S1. original images of WB.pdf]
